# Tocilizumab in the treatment of twelve cases with aa amyloidosis secondary to familial mediterranean fever

**DOI:** 10.1186/s13023-017-0642-0

**Published:** 2017-05-30

**Authors:** Serdal Ugurlu, Aysa Hacioglu, Yasaman Adibnia, Vedat Hamuryudan, Huri Ozdogan

**Affiliations:** Division of Rheumatology, Department of Medicine, Cerrahpasa Medical Faculty, University of Istanbul, Istanbul, Turkey

**Keywords:** Familial Mediterranian fever, Tocilizumab, AA amyloidosis

## Abstract

**Background:**

There is no established treatment of AA amyloidosis, a long-term complication of various chronic inflammatory diseases associated with increased mortality, such as familial Mediterranian fever (FMF). Recently there are few reports pointing out that tocilizumab(TCZ), an anti IL-6 agent may be effective in AA amyloidosis resistant to conventional treatments. We report our data on the effect of TCZ in patients with FMF complicated with AA amyloidosis.

**Methods:**

FMF patients with histologically proven AA amyloidosis, treated with TCZ (8 mg/kg per month) were followed monthly and the changes in creatinine, creatinine clearance, the amount of 24-hour urinary protein, erythrocyte sedimentation rate (ESR) and C-reactive protein (CRP) were noted throughout the treatment period. Adverse effects of the treatment were closely monitored.

**Results:**

TCZ was given to 12 patients (6 F, 6 M) who also continued to receive colchicine (1.9 ± 0.4 mg/day). Coexisting diseases were ankylosing spondylitis(4) and Crohn’s disease(1). The mean age was 35.2 ± 10.0 years and the mean follow-up on TCZ was 17.5 ± 14.7 months. The renal functions remained stable (mean creatinine from 1.1 ± 0.9 mg/dl to 1.0 ± 0.6 mg/dl), while a significant decrease in acute phase response (the mean CRP from 18.1 ± 19.5 mg/L to 5.8 ± 7.1 mg/L and ESR from 48.7 ± 31.0 mm/h to 28.7 ± 28.3 mm/h) was observed and the mean 24-hour urinary protein excretion reduced from 6537.6 ± 6526.0 mg/dl to 4745.5 ± 5462.7 mg/dl. Two patients whose renal functions were impaired prior to TCZ therapy improved significantly on this regimen. No infusion reaction was observed. None of the patients experienced any FMF attack under TCZ treatment with the exception of 2, one of whom had less frequent attacks while the other had episodes of erysipelas-like erythema.

**Conclusıon:**

Tocilizumab improved the acute phase response and the renal function in this group of patients and was generally well tolerated. Besides improving the renal function TCZ seemed to control the recurrence of FMF attacks too. Further studies are warrented to test the efficacy and safety of TCZ in AA amyloidosis secondary to FMF as well as other inflammatory conditions.

## Background

Familial Mediterranean Fever (FMF) is an autosomal recessive autoinflammatory disease characterized by recurrent attacks of fever and serositis, prevalent among eastern Mediterranean populations. Life-long, daily colchicine treatment prevents the recurrence of inflammatory attacks and also the development of AA amyloidosis, which is the most devastating complication of the disease related with increased mortality [1]. Development of AA amyloidosis in a compliant patient on regular prophylactic dose of colchicine is extremely rare. However poor compliance is common and intolerance due to side effects may render the patient from receiving the proper dose that will protect from amyloidosis [2].

FMF is the most common cause of AA amyloidosis in Turkey with an overall frequency of 1-2/1000 and amyloidosis is diagnosed in about one tenth of this population [3, 4].

Although a number of agents have been considered, there is no established treatment of AA amyloidosis. IL-6 is one of the pro-inflammatory cytokines playing a critical role in the induction of SAA genes, thus inhibition of IL-6 results in dramatic suppression of SAA [5, 6]. Recently several case reports have been published showing that tocilizumab (TCZ), a humanized monoclonal anti IL-6 receptor antibody, was effective in the treatment of amyloidosis secondary to various rheumatic diseases. It binds to soluble and membrane-bound IL-6 receptors and down regulates the synthesis of IL-6 with significant decrease in SAA levels [7, 8].

Here we report our experience with TCZ in the treatment of 12 FMF patients complicated with AA amyloidosis.

## Methods

In this case series, 14 patients recieved TCZ with the diagnosis of FMF related AA amyloidosis. Only the results of 12 are given here because of suspect diagnosis of FMF in one, and the discontinuation of TCZ after hypertensive attack observed right after the first infusionin the other patient. All 12 patients with biopsy-proven FMF amyloidosis were regular attendees of the dedicated FMF clinic in Cerrahpasa Medical Faculty. They fullfilled the Tel-Hashomer criteria for FMF [9].

Fever (11 patients), abdominal pain (11 patients) and arthralgia (11 patients) were the most frequent symptoms experienced by our patient group during the attacks. Other generally less frequent attack features like arthritis (11 patients) and myalgia (ten patients) were also frequent in our patient group.

Four patients had concomittant ankylosing spondilitis (AS), diagnosed according to the modified New York criteria [10] and one of them also had Crohn’s disease.

The diagnosis of amyloidosis was confirmed by detecting amyloid deposits in the tissues obtained either from rectum (two patients) or kidney (ten patients). The specimens were stained with Congo red and evaluated for yellow-green birefringence by polarizing microscope.

The indications for TCZ treatment were high acute phase response during attack-free periods and deterioration of renal and/or gastrointestinal functions due to amyloidosis on maximum tolerated dose of colchicine. Patients with end stage renal disease (ESRD) on dialysis were excluded. All of the patients continued daily prophylactic dose of colchicine except one, who experienced serious diarrhea and vomiting on this medication. The patients were treated with TCZ 8 mg/kg body weight/month. No premedication was given.

Each patient was monitored monthly for the following parameters: creatinine, creatinine clearance, the amount of 24-hour urinary protein, erythrocyte sedimentation rate (ESR) and C-reactive protein (CRP). Patients were questioned for the recurrence of FMF attacks and were closely monitored for adverse and side effects of the treatment.

For statistical purposes only the measurements obtained before starting TCZ treatment and at the last observation were considered.

In addition, we searched the PubMed using keywords ‘tocilizumab’ and ‘familial Mediterranean fever’ and compared the resulting five studies in Table 1.

**Table 1:** Comparison of the studies on the use of Tocilizumab treatment in FMF patients

	Fujikawa et al. (2013) [33]	Serelis et al. (2015) [34]	Hamanoue et al. (2015) [18]	Yilmaz et al. (2015) [19]	Umeda et al. (2015) [35]	Our cases
Total number of patients	1	1	1	11	1	12
Adult/children	1/0	1/0	1/0	11/0	1/0	12/0
Duration of Tocilizumab treatment, mean (months)	Not specified	48	41	57	9	18
Gene analysis						
M694V/M694V				7		4
M694V/M680I				2		1
M694I/- heterozygous	1					
Other		Not specified	1	2	1	7
Indications for Tocilizumab treatment						
Amyloidosis		1	1	11		12
Frequent attacks (fever, serositis)	1	1	1		1	
Arthritis/spondylitis		1	1			
Protracted myalgia					1	
Henoch-Schonlein purpura						
Colchicine intolerance/side effect						
Effect of Tocilizumab during follow up						
No attacks	1		Unknown	8	1	10
Decrease number of attacks		1				1
No response						1
Proteinuria in patients with amyloidosis		decreased	decreased	Decreased in 7		9
Side effects	None	Unknown	Unknown	Elevation of liver enzymes, mild thrombocytopenia	Not specified	Transient diplopia, deterioration in hypertension

### Statistical analysis

Continuous variables with more or less normal distribution were expressed as mean ± S.D.

All tests were performed using version 13.0 of SPSS software for Windows (SPSS Inc, Chicago, IL, USA).

## Results

The demographic characteristics and treatment details of 12 patients with the definite diagnosis of FMF and biopsy proven AA amyloidosis who received TCZ are given in Tables 2 and 3. The mean age of the patients was 35.2 ± 10.0, the mean duration of FMF was 15.0 ± 9.2 and of amyloidosis was 3.9 ± 4.8 years. The mean maximum dose of colchicine before TCZ therapy was 1.9 ± 0.4 mg/day.

**Table 2:** Demographic characteristics of the patients

Demographic characteristics (*n* = 12)	
Age (years), mean ± SD	34.2 ± 10.3
Sex, M(%)/F (%)	6 (%50.0)/6 (%50.0)
Disease duration (years), mean ± SD	6.43 ± 6.90
Duration of amyloidosis (years), mean ± SD	3.9 ± 4.8
TCZ therapy duration (months), mean ± SD	17.5 ± 14.7
Number of applied TCZ doses, mean ± SD	15.3 ± 12.1
Co-existing diseases	n (%)
Ankylosing spondylitis	4 (%33.3)
Crohn’s disease	1 (%8.3)

**Table 3:** Values obtained before TCZ initiation and after the last infusion for each patient

Patients	Age (year)	Sex	Comorbid diseases	Disease duration (year)	Amyloidosis duration (year)	Number of applieddoses	Previous biotherapies	Creatinine before treatment (mg/dl)	Creatinine after treatment (mg/dl)	GFR before treatment (ml/min)	GFR after treatment (ml/min)	Proteinuria before treatment (mg/day)	Proteinuria after treatment (mg/day)	CRP before treatment (0–5 mg/L)	CRP after treatment (0–5 mg/L)	Sedimentation before treatment (mm/h)	Sedimentation after treatment (mm/h)	MEFV gene mutation
1	36	M	0	1	1	6	-	3.24	2.4	37.52	45.14	12000	2072	1.5	0.3	32	7	M694V/R761H compound heterozygote
2	44	M	0	27	1	5	Anakinra	2.58	1.86	39.94	59	23677	14962	11.5	2.73	107	43	M694V homozygote
3	45	M	AS	28	0.58	31	Canakinumab	1.28	1.12	86.83	98.72	4725	4462.5	25.5	13.23	32	8	M694Vhomozygote-R202Q homozygote
4	47	F	0	14	0.41	31	-	0.8	0.83	80.84	82.76	2100	1440	14.7	0.31	24	1	M694Vhomozygote-R202Q homozygote
5	23	M	0	15	1	4	-	0.69	0.71	184.9	192.16	3038.5	1392	15.7	14	20	8	
6	35	M	AS	28	10	28	Infliximab	1.18	1.4	92	81.71	1715.6	2720	13.5	0.72	7	3	M694Vhomozygote-R202Q homozygote
7	41	F	0	17	16	13	Cyclophosphamide	0.72	0.65	116	106.2	3000	1950	9.2	10.14	75	40	M694V homozygote
8	39	F	CROHN DISEASE, AS	7	7	32	Infliximab, Cyclophosphamide, Anakinra, Canakinumab	0.43	0.55	136.6	96	1588	740	9.8	21.5	50	40	M694V homozygote
9	24	F	0	20	3	4	Anakinra	0.4	0.36	166.6	225.89	6018	4707	69.7	3.5	69	61	M694V homozygote
10	22	F	0	8	1	20	-	0.39	0.5	175.02	133	1827	80	40	0.42	89	3	MEFV −/−
11	45	F	AS	8	3	6	Anakinra, Infliximab, Etanercept, Canakinumab	0.8	0.93	80.7	54.3	7061.76	5680	4.4	1.6	56	91	M680Iheterozygote-M694Vheterozygote
12	21	M	0	7	3	4	Anakinra	0.83	0.85	143.6	132.22	11700	16740	1.31	1.42	23	29	M694V/N heterozygote

The mean duration of TCZ therapy was 17.5 ± 14.7 months and the mean number of infusions was 14.2 ± 12.3, the range being 4–32.

Symptoms of the patients during attacks were fever and abdominal pain in 11 (91.7%), arthritis in 9 (75.0%), erysipelas-like erythema in 4 (33.3%) and pleuritis in 2 (16.7%).

Eight patients had received several DMARDs or other biological agents before TCZ either for amyloidosis or associated diseases. Anakinra was given to five, canakinumab to three, infliximab to three, cyclophosphamide to two, etanercept to one, sulfasalazine to two and azathioprine to one patient(Table 3). The reasons for terminating these agents wereeither ineffectiveness or allergic reactions.

The renal functions remained stable (the mean creatinine from 1.1 ± 0.9 mg/dl to 1.0 ± 0.6 mg/dl and the mean GFR from 111.7 ± 50.1 ml/min to 108.9 ± 54.8 ml/min) and the mean 24-hour urinary protein excretion reduced from 6537.6 ± 6526.0 mg/dl to 4745.5 ± 5462.7 mg/dl, while a significant decrease in acute phase response (the mean CRP from 18.1 ± 19.5 mg/L to 5.8 ± 7.1 mg/L and the mean ESR from 48.7 ± 31.0 mm/h to 27.8 ± 28.3 mm/h) was observed.

Glomerular filtration rate (GFR) was below 50 ml/min in two patients. After a mean follow up period of 6.5 months on TCZ therapy, the creatinine decreased from 3.2 mg/dl to 2.4 mg/dl in one patient and from 2.6 mg/dl to 1.9 mg/dl in the other, while GFR increased from 37.5 ml/min to 45.1 ml/min and from 39.9 ml/min to 59.0 ml/min, respectively. Also 24-hour proteinuria levels decreased from 12000 mg/day to 2072 mg/day and from 23677 mg/day to 14962 mg/day, respectively. There was also an improvement in CRP (from 1.5 mg/L to 0.3 mg/L and from 11.5 mg/L to 2.7 mg/L, respectively (referance range for CRP: 0–5 mg/L)) and sedimentation rates (from 32 mm/h to 7 mm/h and from 107 mm/h to 43 mm/h, respectively).

The remaining ten patients had GFR over 50 ml/min and the mean duration of TCZ therapy was 21.5 ± 14.5 months. The mean creatinine and GFR of this group remained nearly the same (from 0.8 ± 0.3 mg/dl to 0.8 ± 0.3 mg/dl and from 126.3 ± 40.6 ml/min to 120.3 ± 52.8 ml/min, respectively). The mean 24-hour urine protein level decreased from 4277.4 ± 3228.1 mg/day to 3991.2 ± 4842.3 mg/day. A significant decrease was observed in the mean CRP (from 20.4 ± 20.6 mg/Lto 6.3 ± 5.9 mg/L) and ESR levels (from 44.5 ± 27.3 mm/h to 28.4 ± 30.0 mm/h).

To see whether the group with a comorbidity (*n* = 4) had an additional burden of inflammation we compared the acute phase response of this group with only FMF patients (*n* = 8). The initial CRP and ESR levels before initiation of TCZ treatment were lower in the first group (CRP13.3 ± 9.0 vs 20.5 ± 23.3 mg/L; ESR 36.3 ± 22.0 vs 54.9 ± 34.2 mm/h) as the initial 24- hour proteinuria compared to the eight patients without any comorbidity (3772.6 ± 2628.6 vs 7920.1 ± 7576.9 mg/d). In addition the response to treatment with regard to these variables were comparable. Table 4 represents the renal funcions and acute phase responses comparing the whole group with AA-FMF-only group and AA-FMF-coexisting diseases group (Table 4). These observations point out that there was no increased sign of inflammatoin in the group with a coexisting disease at the time they were exposed to IL-6 blockade.

**Table 4:** Comparison of the groups on the basis of renal functions and acute phase parameters

	All group(*n* = 12)		AA + FMF(*n* = 8)		AA + FMF + co-existing diseases(*n* = 4)	
	Before treatment	After treatment	Before treatment	After treatment	Before treatment	After treatment
Creatinine (mg/dl) (Female: 0.5–0.9; Male:0.7–1.2)	1.1 ± 0.9	1.0 ± 0.6	1.2 ± 1.1	1.0 ± 0.7	0.9 ± 0.4	1.0 ± 0.4
GFR (ml/min) (71–151)	111.7 ± 50.1	108.9 ± 54.8	118.1 ± 59.4	122.1 ± 62.8	99.0 ± 25.5	82.7 ± 20.4
Proteinuria (mg/day)	6537.6 ± 6526.0	4745.5 ± 5462.7	7920.1 ± 7576.9	5417.9 ± 6585.0	3772.6 ± 2628.7	3400.6 ± 2149.8
CRP (0–5 mg/L)	18.1 ± 19.5	5.8 ± 7.1	20.5 ± 23.3	4.1 ± 5.2	13.3 ± 9.0	9.3 ± 10.0
Sedimentation (0–20 mm/h)	48.7 ± 31.0	27.8 ± 28.3	54.9 ± 34.2	24.0 ± 22.4	36.3 ± 22.0	35.5 ± 40.5

No FMF attack was observed in 10 of the 12 patients, while one, who also had diagnosis of AS, experienced less frequent and mild attacks. There was only one patient who had recurrent attacks of erysipelas-like erythema (ELE) under TCZ, therefore the treatment was switched to canakinumab.

The interval between the TCZ infusions was increased to 2 months in one patient due to significant improvment in her renal functions, but proteinuria and acute phase reactants increased after the first bimonthly regimen and the therapy was switched back to monthly infusions. Similarly, one other patient whose renal function had improved on TCZ monthly for nearly 2.5 years, deteriorated rapidly after skipping only 2 doses of TCZ because of a respiratory tract infection. She ended up with ESRD and is on hemodialysis.

One patient with starting GFR below 50 ml/min who had benefitted from TCZ therapy was lost to follow up after the 3rd dose. Two years later when he was back to the clinic with a dramatic increase in his 24-hour proteinuria (21000 mg/day), TCZ was reinstituted. Because there was no improvement in his renal function tests and because his acute phase response was in progress after the 2nd dose, the therapy was switched to canakinumab.

Patient with co-existing AS complained of short-lived inflammatory back pain twice which did not necessitate termination of TCZ treatment.

### Side effects

One patient experienced transient diplopia after the 6th dose. Her neurological examination and cranial MRI were normal. TCZ was terminated and she rapidly developed ESRD thereafter. One other patient with normal renal functions who already had a diagnosis of essential hypertension and coronary artery bypass operation 4 months before the initiation of TCZ, experienced an increase in her blood pressure after the 13th dose and the therapy was switched to canakinumab.

Treatment was stopped in one patient due to hypertensive encephalopathy which developed 1 week after the first dose of TCZ, therefore he was not included in this serie. However later it became clear that the patient was an addict of a synthetic cannabinoid drug which may have contributed to this clinical picture.

None of the patients had opportunistic infections during the therapy. One patient had a non-complicated urinary tract infection during which TCZ dose was skipped and the patient received antibiotics. Another one had a respiratory tract infection which responded well to therapy but after omitting 2 doses of TCZ the patient’s renal functions deteriorated.

Biochemical analysis were routinely performed before every dose of TCZ and there were no elevations of transaminases during the therapy. Also we did not observe any pathology in the blood count parameters.

None of the patients developed infusion reactions.

## Discussion

Although a number of agents like azathioprine, anti TNF-alpha agents, eprodisate, anti IL-1 antagonists have been considered, there is no established treatment of AA amyloidosis by today [11–14]. Recently beneficial effect of TCZ, an anti IL-6 agent in the treatment of amyloidosis secondary to JIA has been reported [5]. This was followed by other cases of RA, Behcet’s Disease and FMF complicated with AA amyloidosis and a case series of 11 patients with FMF amyloidosis treated with TCZ [8, 15–19]. All reported an overall improvement of renal function, decrease in proteinuria and acute phase response.

Serum amyloid A protein (SAA) is an acute-phase reactant mainly synthesized by the liver, which is overproduced during inflammatory conditions in response to various cytokines [20] and which is the precursor of AA fibrils leading to secondary amyloidosis [21]. Supression of SAA protein production by treatment of the underlying inflammatory disease resulted in regression of amyloid deposition in organs and in a better outcome [22]. IL-6 is one of the mediators known to be responsible in the pathogenesis of the FMF. [23–25] It also induces synthesis of serum amyloid A (SAA) in hepatocytes and its inhibition is postulated to be effective in the treatment of AA amyloidosis.

SAA, ESR and CRP are good indicators of disease activity and response to treatment of the underlying disease that cause amyloidosis [26]. It has been shown by Lachmann that elevated levels of SAA was related with an increased risk of amyloidosis [27]. However the data is scarce, a positive correlation between these markers has been reported [28]. Compared to routinly tested CRP and ESR, SAA is not used widespread in daily practice. Here we report only the results of CRP and ESR, because not all patients have been tested for SAA at each visit.

It is expected that under IL-6 inhibition CRP levels will be low and thus it may not be an ideal indicator of underlying inflammation. However, it is well established that TCZ also inhibits the production of SAA. As SAA is the amyloid precursor protein, reduction in circulating SAA by TCZ will suppress further amyloid load and may allow amyloid regression in some cases. Thus, whilst TCZ may not treat the underlying condition, it will suppress production of amyloid precursor protein and may be a reasonable treatment option in this setting.

Creatinine clearance and proteinuria are good parameters used in the follow up of renal function in patients with amyloidosis. Overall the mean creatinine clearance remained stable while the mean 24-hour proteinuria and acute phase response decreased. In two patients with GFR below 50 ml/min before the initiation of TCZ, creatinine clearence, proteinuria and acute phase reactants improved significantly. One interesting observation was that renal function deterioriated rapidly in two patients after transient discontinuation of treatment for side effects and in one after increasing the infusion interval from one to 2 months for good response. The rapid worsening of renal function after cessation of TCZ treatment in patients with significant beneficial response may suggest that TCZ do not have a direct effect on the amyloid burden of the end organs in these patients.

Among the study group 3 had coexisting AS and one had Crohn’s disease. At the initiation of TCZ treatment, all four were in remission with regard to their co-morbidities and did not require any additional therapy. It has been reported that TCZ is not effective in the treatment of AS and one of these four patients experienced mild inflammatory back pain twice. Also in sound with studies presenting the effectiveness of TCZ on Crohn’s disease [29–31], no exacerbation was detected in the patient with associated inflammatory bowel disease.

We observed hypertension in two patients. Although TCZ’s hypertensive effect is well known, we could not rule out the contribution of cannabinoid use in one. The transient diplopia that we observed in another patient could be a side effect of TCZ, however diplopia related to this medication has not been reported previously.

In a trial published in Germany, TCZ was given to five colchicine resistant FMF patients without amyloidosis; three of them improved while one was stable and the other one had infusion reactions [32]. We observed no attacks in ten patients and significant decrease and nonresponse in one each. However it has been shown that IL-6 is involved in the pathogenesis of the FMF [24, 25], the data is not sufficient to conclude that TCZ is effective in controlling the FMF attacks.

We were not able to screen SAA levels because it is not routinly tested in our laboratory, which we feel is the main limitation of this study.

## Conclusion

TCZ may be an alternative in the treatment of FMF patients with AA amyloidosis who are resistant/intolerant to colchicine. It is well tolerated and has an acceptable adverse effect profile. TCZ was effective in controlling not only the signs related to amyloidosis but also the FMF attacks. Thus TCZ may be another treatment option besides anti-IL-1 approach even for colchicum resistant FMF patients without amyloidosis. One important point is that the patients should be followed closely for a rapid worsening of renal function after stopping or increasing the infusion interval of TCZ treatment. To conclude, TCZ seems to be an effective treatment option in patients with AA amyloidosis with few side effects.

## Abbreviatıons

AS: Ankylosing spondilitis
CRP: C-reactive protein
DMARD: Disease-modifying antirheumatic drugs
ELE: Erysipelas-like erythema
ESR: Erythrocyte sedimentation rate
ESRD: End stage renal disease
FMF: Familial Mediterranian fever (FMF)
GFR: Glomerular filtration rate
IL-1: Interleukin-1
IL-6: Interleukin-6
RA: Rheumatoid arthritis
SAA: Serum amyloid A
TCZ: Tocilizumab
TNF-alpha: Tumor necrosis factor-alpha

## References

[1] Ozdogan H, Ugurlu S. Familial Mediterranean Fever. Switzerland: Springer International Publishing: 2015.Chapter 9,The Emerging Treatments in Familial Mediterranean :p.137-158.

[2] Melikoglu MA, Senel K. Non-response to colchicine in familial Mediterranean fever should be identified accurately. Int J Rheum Dis. 2014. doi: 10.1111/1756-185X.12374. [Epub ahead of print] 10.1111/1756-185X.12374 24702830

[3] Kasifoglu T, Bilge SY, Sari I, Solmaz D, Senel S, Emmungil H (2014). Amyloidosis andits related factors in Turkish patients with familial Mediterranean fever: amulticentre study. Rheumatology (Oxford).

[4] Tunca M, Akar S, Onen F, Ozdogan H, Kasapcopur O, Yalcinkaya F (2005). Familial Mediterranean fever (FMF) in Turkey: results of a nationwide multicenter study. Medicine (Baltimore).

[5] Okuda Y, Takasugi K (2006). Successful use of a humanized anti-interleukin-6 receptor antibody, tocilizumab, to treat amyloid A amyloidosis complicating juvenile idiopathic arthritis. Arthritis Rheum.

[6] Hagihara K, Nishikawa T, Isobe T, Song J, Sugamata Y, Yoshizaki K (2004). IL-6 plays a critical role in the synergistic induction of human serum amyloid A (SAA) gene when stimulated with proinflammatory cytokines as analyzed with an SAA isoform real-time quantitative RT-PCR assay system. Biochem Biophys Res Commun.

[7] Hakala M, Immonen K, Korpela M, Vasala M, Kauppi MJ (2013). Good medium-term efficacy of tocilizumab in DMARD and anti-TNF-α therapy resistant reactive amyloidosis. Ann Rheum Dis.

[8] Miyagawa I, Nakayamada S, Saito K, Hanami K, Nawata M, Sawamukai N (2014). Study on the safety and efficacy of tocilizumab, an anti-IL-6 receptor antibody, in patients with rheumatoid arthritis complicated with AA amyloidosis. Mod Rheumatol.

[9] Livneh A, Langevitz P, Zemer D, Zaks N, Kees S, Lidar T (1997). Criteria for the diagnosis of familial Mediterranean fever. Arthritis Rheum.

[10] Van der Linden S, Valkenburg HA, Cats A (1984). Evaluation of diagnostic criteria for ankylosing spondylitis. A proposal for modification of the New York criteria. Arthritis Rheum.

[11] Fernández-Nebro A, Tomero E, Ortiz-Santamaría V, Castro MC, Olivé A, de Haro M (2005). Treatment of rheumatic inflammatory disease in 25 patients with secondary amyloidosis using tumor necrosis factor alpha antagonists. Am J Med.

[12] Dember LM, Hawkins PN, Hazenberg BP, Gorevic PD, Merlini G, Butrimiene I (2007). Eprodisate for the treatment of renal disease in AA amyloidosis. N Engl J Med.

[13] Kuroda T, Wada Y, Kobayashi D, Murakami S, Sakai T, Hirose S (2009). Effective anti-TNF-alpha therapy can induce rapid resolution and sustained decrease of gastroduodenal mucosal amyloid deposits in reactive amyloidosis associated with rheumatoid arthritis. J Rheumatol Nov.

[14] Özçakar ZB, Özdel S, Yılmaz S, Kurt-Şükür ED, Ekim M, Yalçınkaya F (2016). Anti-IL-1 treatment in familial Mediterranean fever and related amyloidosis. Clin Rheumatol.

[15] Vinicki JP, De Rosa G, Laborde HA (2013). Renal amyloidosis secondary to rheumatoid arthritis: remission of proteinuria and renal function improvement with tocilizumab. J Clin Rheumatol.

[16] Matsui M, Okayama S, Tsushima H, Samejima K, Kanki T, Hasegawa A (2014). Therapeutic benefits of tocilizumab vary in different organs of a patient with AA amyloidosis. Case Rep Nephrol.

[17] Redondo-Pachón MD, Enríquez R, Sirvent AE, Andrada E, Noguera-Pons R, Millán I (2013). Tocilizumab treatment for nephrotic syndrome due to amyloidosis in Behcet’s disease. Ren Fail.

[18] Hamanoue S, Suwabe T, Hoshino J, Sumida K, Mise K, Hayami N (2016). Successful treatment with humanized anti-interleukin-6 receptor antibody (tocilizumab) in a case of AA amyloidosis complicated by familial Mediterranean fever. Mod Rheumatol.

[19] Yilmaz S, Cinar M, Simsek I, Erdem H, Pay S (2015). Tocilizumab in the treatment of patients with AA amyloidosis secondary to familial Mediterranean fever. Rheumatology (Oxford).

[20] Urieli-Shoval S, Linke RP, Matzner Y (2000). Expression and function of serum amyloid A, a major acute-phase protein, in normal and disease states. Curr Opin Hematol.

[21] Merlini G, Bellotti V (2003). Molecular mechanisms of amyloidosis. N Engl J Med.

[22] Gillmore JD, Lovat LB, Persey MR, Pepys MB, Hawkins PN (2001). Amyloid load and clinical outcome in AA amyloidosis in relation to circulating concentration of serum amyloid A protein. Lancet.

[23] Akcan Y, Bayraktar Y, Arslan S, Van Thiel DH, Zerrin BC, Yildiz O (2003). The importance of serial measurements of cytokine levels for the evaluation of their role in pathogenesis in familial Mediterraean fever. Eur J Med Res.

[24] Ben-Zvi I, Livneh A (2011). Chronic inflammation in FMF: markers, risk factors, outcomes and therapy. Nat Rev Rheumatol.

[25] Manukyan GP, Ghazaryan KA, Ktsoyan ZA, Tatyan MV, Khachatryan ZA, Hakobyan GS (2008). Cytokine profile of Armenian patients with Familial Mediterranean fever. Clin Biochem.

[26] Yalçinkaya F, Cakar N, Acar B, Tutar E, Güriz H, Elhan AH (2007). The value of the levels of acute phase reactants for the prediction of familial Mediterranean fever associated amyloidosis: a case control study. Rheumatol Int.

[27] Lachmann HJ, Goodman HJ, Gilbertson JA, Gallimore JR, Sabin CA, Gillmore JD (2007). Natural history and outcome in systemic AA amyloidosis. N Engl J Med.

[28] Lachmann HJ, Sengül B, Yavuzşen TU, Booth DR, Booth SE, Bybee A (2006). Clinical and subclinical inflammation in patients with familial Mediterranean fever and in heterozygous carriers of MEFV mutations. Rheumatology (Oxford).

[29] Cañas-Ventura A, Rodríguez E, Andreu M, Márquez L (2013). Tocilizumab in amyloidosis-associated kidney disease secondary to inflammatory bowel diseases. Dig Dis Sci.

[30] Ito H, Takazoe M, Fukuda Y, Hibi T, Kusugami K, Andoh A, Matsumoto T (2004). A pilot randomized trial of a human anti-interleukin-6 receptor monoclonal antibody in active Crohn’s disease. Gastroenterology.

[31] Korzenik JR, Podolsky DK (2006). Evolving knowledge and therapy of inflammatory bowel disease. Nat Rev Drug Discov.

[32] Stein N, Witt M, Baeuerle M, Proft F, Schulze-Koops H, Gruenke M (2012). Familial mediterranean fever: inhibition of IL-6 signalling as a New therapeutic option in a frequent autoinflammatory syndrome. [abstract]. Arthritis Rheum.

[33] Fujikawa K, Migita K, Tsukada T, Umeda M, Nonaka F, Kawakami A, Eguchi K (2013). Interleukin-6 targeting therapy in familial Mediterranean fever. Clin Exp Rheumatol.

[34] Serelis J, Christaki S, Skopouli FN (2015). Remission of nephrotic syndrome due to AA-amyloidosis, complicating familiar Mediterranean fever, with tocilizumab. Clin Exp Rheumatol.

[35] Umeda M, Aramaki T, Fujikawa K, Iwamoto N, Ichinose K, Terada K, Takeo G, Yonemitsu N, Ueki Y, Migita K, Kawakami A. Tocilizumab is effective in a familial Mediterranean fever patient complicated with histologically proven recurrent fasciitis and myositis. Int J Rheum Dis. 2015 Oct 20. doi: 10.1111/1756-185X.12776. [Epub ahead of print] 10.1111/1756-185X.12776 26481326

